# Profit distribution and managers’ behaviour in vertical integrated medical delivery systems – an experimental economics study

**DOI:** 10.1186/s12913-020-05467-0

**Published:** 2020-07-02

**Authors:** Yunque Bo, Miaojie Qi, Siyu Liu, Jiyu Cui, Youli Han

**Affiliations:** 1 Department of Health Management and Policy, School of Public Health, Capital Medical University, NO 10, Xi Toutiao Rd Youanmenwai District, Beijing, 100069 China; 2 Tianjin Health Development Research Center, Tianjin, China; 3 School of Health Humanities, Capital Medical University, Beijing, China

**Keywords:** Medical delivery system, Vertical integration, Profit distribution, Manager behaviour, Experimental economics

## Abstract

**Background:**

The vertical integration of medical delivery systems (VIMDSs) is a reform direction both in China and worldwide. We conducted a controlled economic experiment to explore decision-making by managers of medical institutions with respect to profits and what influences the distribution mechanism in VIMDSs.

**Methods:**

Students and hospital staff were recruited to make choices regarding the role of directors of institutions. z-Tree software was used to design the experimental program. Ninety-six subjects participated in the experiment. We gathered 479 valid contracts.

**Results:**

Of the subjects, 66.39% chose flexible contracts. The median of the bidding distribution rate to community health service centres of all auctions was 18.50%. The final distribution rate was approximately 3 percentage points higher than the bidding distribution rate. The median effort level was 9.00. There was a significant correlation between the improvement rate and the choice of effort level (*P<0.05*) in flexible contracts.

**Conclusions:**

Hospital managers have a preference for flexible contracts because of uncertainty in the medical system. A community health service centre director may behave perfunctorily by engaging in shading in the integration. Flexible contracts and sharing rates beyond the participants’ expectations motivate managers to engage in more cooperative behaviours.

## Background

The fragmentation of medical care has become one of the main reasons for the inefficiency of medical delivery systems in many countries (WHO 2009), which may subsequently raise health care expenditure and expand the scale of hospitals. To solve these problems, many countries have begun exploring the integration of health care systems [1–3]. The vertical integration of medical delivery systems (VIMDSs), which involves integrating hospitals and community health service centres within a district to deliver continuous medical care, is one of the directions of healthcare reform in China [4]. Managers of medical institutions attach importance to the implementation and efficiency of vertically integrated institutions. Related studies [5, 6] show that profit distribution through integration among different institutions is a core problem. Managers’ understanding of and attitude towards profit distribution is an important factor that influences their pursuit of the goal of integrating medical delivery systems. Therefore, it is necessary to ascertain the relationship between profit distribution and managers’ behaviour in VIMDSs.

Our discussion of the relationship between profit distribution and managers’ behaviour in VIMDSs is based on incomplete contract theory. Grossman & Hart (1986) [7], Hart & Moore (1990) [8], and Hart (1995) (GMH) [9] developed the incomplete contract theory of vertical integration. This theory provided a framework for analysing the cost and profits of ownership. On this basis, incomplete contract theory explores the effects on vertical integration. Hart and Moore [10] introduced behavioural assumptions to test incomplete contract theory. They proposed that a contract provides a reference point for managers’ feeling of entitlement and defined a concept, “nature state”, that represented the uncertainties of future political, economic, and other conditions. In their model, trading partners meet in a competitive market before they enter a bilateral relationship. They sign an incomplete contract, and if the parties fail to obtain the rights stipulated in the contract, they can always contribute a perfunctory performance instead of a consummate performance (they are reluctant to exert maximum effort in cooperating) [11]. Hart and Moore considered “perfunctory performance” to be “shading”. They assumed that contracts provide reference points for entitlements, which implies an interesting trade-off between contractual rigidity and flexibility [12]. A flexible contract allows the trading parties to adjust the terms of the contract according to nature states, but a rigid contract does not. However, the multiplicity of outcomes in flexible contracts makes it possible for the parties to engage in shading. Fehr, Hart, and Zehnder [13, 14] introduced an experimental method to validate reference point theory. Experimental economics is a method of studying decision-making behaviour by simulating realistic environments to examine the choices of decision-makers. Like real-world data, experimental results are an important way to provide evidence [15, 16]. Economic experiments have been used successfully to explore the effects of vertical integration in nonhealth fields [12–14] and decision-making behaviours in health fields [17–23]. In VIMDSs, the decision-making behaviour of medical institution managers has not been verified by experimental methods; therefore, we designed such an experiment. The experiments were programmed and conducted using z-Tree [24].

A VIMDS is a contractual relationship between hospitals and community health service centres. The prevalence of uncertainty in health care is a distinctive feature of health economics [25]. In VIMDSs, it is difficult to determine in advance the types of services, in addition to their content, quality, and cost, that should be provided among different medical institutions, or the cost of determining them may be too great. It is difficult for the government to stipulate the contents that different medical institutions should provide and the specifications of referral through policy (as in a complete contract). Therefore, the contract is incomplete.

According to incomplete contract theory and based specifically on its further development — the theory of contracts as reference points — the managers of different medical institutions sign contracts for the method of distribution and the rate of future integrated profits. The contracts provide reference points. Then, the community health service centre managers choose the effort level that they are willing to invest for integration. If they are satisfied with the distribution rate, a consummate performance will be provided instead of shading. Our research is based on the framework of incomplete contract theory. According to the experimental design of Fehr, Hart, and Zehnder (2011) [13] and considering special factors in the field of health, we designed the experiment of this study. We conducted a controlled economic experiment to explore decision-making by managers of medical institutions with respect to profits and what influences the distribution mechanism in VIMDSs.

The purpose of integrating hospitals and community health service centres is to change the phenomenon of disordered treatment so that patients can visit a community health service centre first and then, if a referral is needed, be referred to a hospital. However, hospitals may have fewer patients than before, so they have no incentive to integrate with community health service centres, and the community health service centres may not have the ability to integrate because of capacity limitations. Therefore, governmental agencies should motivate medical institutions by recommending the integration of medical delivery systems. We assumed that the social insurance department can package medical insurance funds for the integrated entity in the form of a global budget. Medical insurance funds that are saved by integration are allocated to the integrated entity. The hospital managers and community health service centre directors need to determine the proportions of the profit distribution. Accordingly, we introduced a controlled economic experiment to analyse how medical institution managers make choices of profit distribution and how their choices influence VIMDSs. Students and professionals were recruited to act as the managers of either hospitals or community health service centres in each experimental session (Ethical Review Number 2018SY86). It is very common for students to be the subject of experiments in economic experiments [13–21]. To test the validity, we also recruited one group of real medical institution managers, called professionals, as subjects. We wanted to answer two research questions. The first one deals with whether the effort level becomes greater as the distribution rate for the community health service centres becomes higher (Research Question 1). The second concerns whether shading behaviours exist in the process of vertical integration (Research Question 2).

## Methods

### Details of the experiment and parameters

Hospital managers and community health service centre directors are involved in VIMDSs. The design of the study was inspired by Fehr, Hart and Zehnder (2011) [13]. In their design, the buyer determines whether he or she wants to conclude a flexible or a rigid contract. A competitive auction determines which seller gets the contract. Then, the buyer provides the price, and the seller chooses the quality of the goods to be provided. Rigid contracts are characterized by a fixed price, but flexible contracts allow for a price range from which the buyer can select a price after the uncertainty about the state of nature has been resolved. In our experiment, the distribution rate was “price”, and the effort level was “quality”. Decisions about the distribution rate and effort level were made independently at each auction.

First, hospital managers chose the contract type (rigid contract or flexible contract). A rigid contract means that the distribution rate cannot be adjusted. The final distribution rate of a flexible contract can be adjusted after the nature state is revealed. Second, the two contracts were chosen randomly in turn for community health service centre directors to bid on, and the directors’ auction bidding distribution rate (*r*_*1*_) represented the percentage of integration profits allocated to community health service centres. The decision-making behaviours in two successive auctions did not interfere with each other. The community health service centre directors had two chances to bid on a contract. Each hospital could work with only one community health service centre, and each community health service centre had the opportunity to work with two hospitals. The reason for this design is that there are more community health service centres than hospitals, so it is competitive for community health service centres to cooperate with hospitals. In each period, 2 successful contracts at most were signed. Then, according to different random nature states (good or bad), as in the design of GMH [7–9], the nature state represented future uncertainty. Hospital managers who chose flexible contracts needed to decide the final distribution rate (*r*_*2*_). Finally, community health service centre directors chose their effort level (*E*). The effort level would ultimately affect the effectiveness of integration. In each period, managers at the two hospitals chose partners in turn. This implies that community health service centre directors have 2 chances to bid for the contract chosen by the hospital manager. In the experiment, the subjects needed to represent hospital managers or community health service centre directors to make decisions. There were 24 subjects in each experimental session; all participants were divided into 6 groups randomly by a computer program. Each group had 2 hospital managers and 2 community health service centre directors. The roles in the experiment were also randomly assigned and were reassigned in each round.

**Fig. 1:**
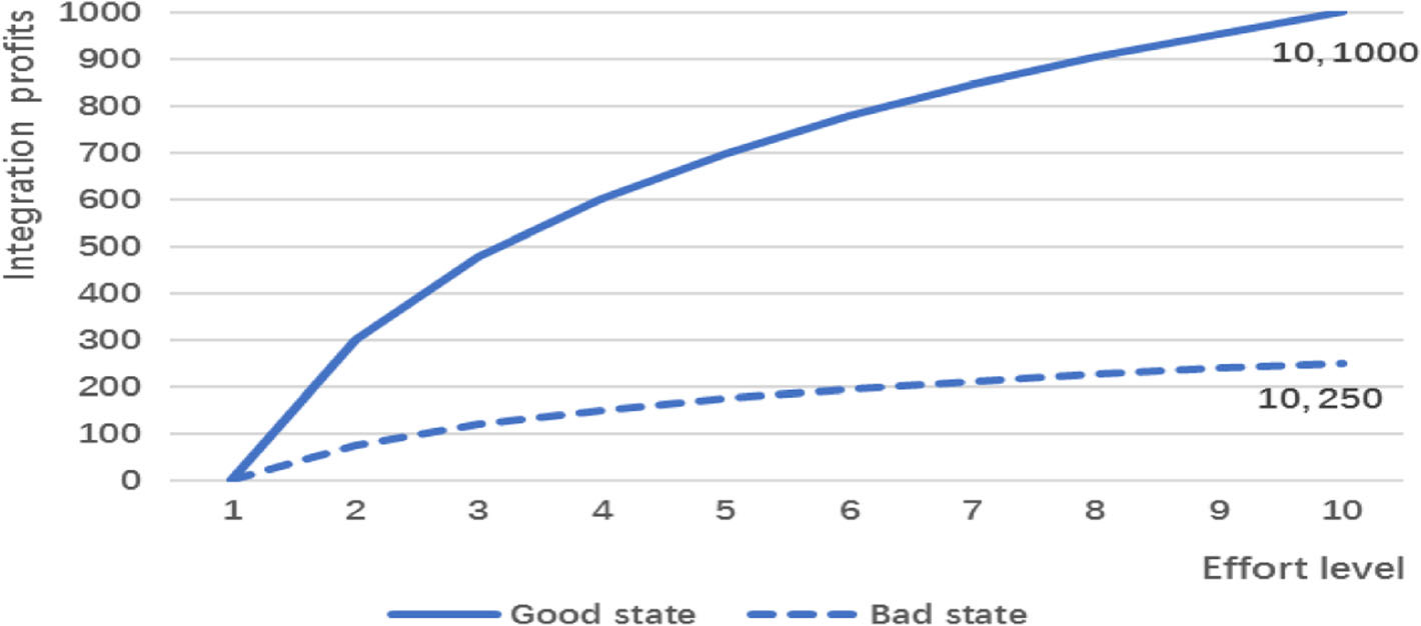
Relationship between effort level and integration profits. This figure shows the relationship between effort level and integration profits in two nature states. Integration profits refers to the increased profit attained through integration; when there is no effort, the integration profit is zero. In the good state, Y* is 1000.00, whereas in the bad state, it is 250.00

It is difficult to estimate the cost data for cooperation in VIMDSs, because there was no relevant real-life evidence. Therefore, according to the relationship between the proportion of treatment in the primary medical institutions and the integration goal, we fitted the relationship between the effort level and the integration benefits (as shown in Fig. 1).

In 2015, the China State Council issued guidance on the construction of the classification and treatment system and proposed that the treatment rate for residents in the county should increase to approximately 90.00% and that the proportion of total treatment provided by primary health care institutions should be greater than or equal to 65.00%.^1^ According to the survey data, the proportion of treatment in primary medical institutions is 32.10%. To achieve the policy goal, community health service centres need to make greater efforts from the lowest level to the highest level. The basic assumption of the experiment was that medical insurance packages are paid to the integrated entity, and the savings are used as compensation for the hospitals and community health service centres. There is a lack of evidence regarding the amount of expense that can be saved by integration, so we provided a virtual number in the experiment and called it integration profit. We assumed different highest profits (Y*) in the two nature states; thus, the profit is Y^*^*log(*E*).

The profit (benefit) calculation formula is as follows:

The rigid contract:

$$ {\displaystyle \begin{array}{c} community\ health\;\mathrm{service}\; centres' benefit:B={r}_1\ast {Y}^{\ast}\ast \mathit{\log}(E)\\ {} hospitals' profits:P=\left(1-{r}_1\right)\ast Y\ast \mathit{\log}(E)\end{array}} $$

The flexible contract:

$$ {\displaystyle \begin{array}{c} community\ health\;\mathrm{service}\; centres' benefit:B={r}_2\ast {Y}^{\ast}\ast \mathit{\log}(E)\\ {} hospitals' profits:P=\left(1-{r}_2\right)\ast Y\ast \mathit{\log}(E)\end{array}} $$

### Experimental procedures

- Step 1: The hospital managers chose contracts.

One hospital manager could choose only one type of contract (either a rigid contract or a flexible contract). Two contracts would be generated within a group.

- Step 2: The community health service centre directors bid for a contract.

One random contract was auctioned to the community health service centre directors. The bidding distribution rate was the proportion of integration profits allocated to the community health service centre. The bidding distribution rate was initially 10.00%, with an upper limit of 40.00%, and it then increased by 0.50% every second over a countdown period of 60 s. Each centre director could press a button to accept the contract before the end of the countdown. The first centre director who pressed the button to accept the rate would obtain the contract. The bidding distribution rate was the final distribution rate for a rigid contract; however, for a flexible contract, it was the lower limit of the final distribution rate.

- Step 3: Determination of the nature state.

A computerized random device determined the nature state for each contract independently. The highest profit was different in the two states. The probabilities of the two nature states were 0.80 and 0.20, respectively.

- Step 4: The hospital managers chose the final distribution rate.

Once the nature state was revealed, the hospital managers who chose flexible contracts determined the final distribution rate. The range of the final distribution rate was from the bidding distribution rate to 60.00%^2^ [26]. The hospital managers who chose rigid contracts had no adjustment options.

- Step 5: The community health service centre directors chose the effort level.

The community health service centre directors observed the states and the final distribution rate that the hospital managers chose. Then, they determined their effort levels. Effort level 1 represented no effort, and effort level 10 represented the greatest effort.

- Step 6: Profit calculation.

After the centre directors’ choices of effort levels were made, the profits were calculated and displayed on the subjects’ screens.

- Step 7: New Round.

A new round began. The participants were reassigned randomly to a new interaction group and went through these steps again. Each experiment had 10 rounds.

### Subjects and payments

We organized 4 experiments for which we recruited 96 subjects. The participants in three experiments were undergraduate and postgraduate students of Capital Medical University who were majoring in health management. They had knowledge of hospital management and a high level of compliance with the experimental design. The remaining subjects, whom we called professionals, were administrative staff from hospitals with 2–8 years of work experience. The students had a relatively steep learning curve. Therefore, after reading the guidelines, they could behave as a reasonable proxy of real managers in a short time. Students may have an advantage in this regard because they have less exposure to confounding external information, which makes our research less biased. Our study focuses on the mechanism of profit distribution and managers’ behaviour. Real managers may consider other factors in making their choices of contract type, distribution rate, effort level and so on. Then, their opportunity cost is lower, and they can participate in multiple repetitive trials.

In each experiment, the subjects were randomly subdivided into different groups before the start of each experiment. All the interactions were anonymous, and the subjects did not know the personal identities of their partners. To ensure that the subjects fully understood the procedures and the payoff consequences of the available actions, each subject was given a detailed set of materials before the experiment started. We provided a payoff according to the choices of the subjects with the purpose of motivating the subjects and controlling the outcome. The minimum remuneration of the student pool was 50 CNY, while that of the professionals was 200 CNY. The total remuneration per subject was the basic pay plus the motivation amount calculated by experimental currency units. The exchange rate between the experimental currency units (“points”) and real money was 100 points = 1 CNY.

### Analysis methods

Z-Tree was used to design the experimental procedures and collect data. The data were exported from z-Tree and sorted in Excel, and SPSS20.0 was used for analysis. Because the data did not conform to the normal distribution, they were presented as the median and quartile. We compared the differences in the subjects’ choices between the two types of contracts using the Mann-Whitney U test and compared the differences between the three types of subjects using the Kruskal-Wallis (K–W) test. We used the Spearman rank test to analyse the relationship between the distribution rates (the bidding distribution rates, the final distribution rate, and the improvement rate) and the effort level. In addition, logistic regression was applied to analyse the effect of different factors on the effort level. All *p*-values < 0.05 were considered statistically significant.

## Results

### Descriptive analysis of choices

The experiment yielded a total of 480 contracts, of which 479 samples were valid and 1 sample was invalid due to streaming. As shown in Table 1, 66.39% of the subjects chose flexible contracts, and 33.61% of the subjects chose rigid contracts. After the hospital managers had chosen the type of contract, the community health service centre directors began to bid for the contracts. The median bidding distribution rate was 18.50%, the rigid contract rate was 17.00%, and the flexible contract rate was 20.00%. There was no significant difference between the two types of contracts (*P = 0.49*).

**Table 1:** The results of choices under the two contract types

	Subgroup	Rigid contract		Flexible contract		Total		
		Median	Quartile	Median	Quartile	Median	Quartile	*P*
**The bidding distribution rate (%)**	Undergraduates	15.00	4.75	13.50	5.50	14.25	14.25	
	Postgraduates	18.25	12.00	20.50	14.00	20.00	20.00	
	Professionals	25.00	16.25	24.00	5.50	24.50	24.50	
	Total	17.00	12.00	20.00	13.00	18.50	18.50	< 0.001
	Observations (%)	161(33.61)		318 (66.39)		479 (100.00)		–
**The final rate (%)**	Undergraduates	–	–	16.00	6.00	–	–	–
	Postgraduates	–	–	25.00	14.00	–	–	–
	Professionals	–	–	28.00	6.50	–	–	–
	Total	–	–	23.00	14.00	–	–	< 0.001
**The improvement rate (%)**	Undergraduates	–	–	2.00	3.25	–	–	–
	Postgraduates	–	–	4.00	6.00	–	–	–
	Professionals	–	–	3.50	6.50	–	–	–
	Total	–	–	3.00	4.63	–	–	0.01
**The effort level**	Undergraduates	10.00	2.00	10.00	1.00	10.00	1.00	
	Postgraduates	8.00	4.00	8.00	3.00	8.00	3.00	
	Professionals	8.00	4.00	9.00	3.00	8.00	4.00	
	Total	8.00	4.00	9.00	3.00	9.00	3.00	< 0.001

After the nature state was determined, the hospital managers chose the final distribution rate in the flexible contract, and the median and quartile ranges were 23.00% and 14.00, respectively. As shown in Table 1, the professionals chose the highest final distribution rate, and the students chose the lowest final distribution rate. The choices of the three groups of subjects differed statistically, with *P* < 0.001. The final distribution rate was higher than the bidding distribution rate, with an average increase of 3 percentage points that we called the improvement rate. The postgraduates chose the highest rate (4.00), followed by the professionals (3.50), and the lowest rate was chosen by the undergraduates (2.00). There was a statistically significant difference in the choices among the three groups of subjects, with *P* = 0.01.

Additionally, as shown in Table 1, the median effort level was 9.00, where the rigid contract was 8.00 and the flexible contract was 9.00. However, there was no significant difference between the two types of contracts *(P = 0.09)*. In the three groups of subjects, the effort level of the undergraduate group was significantly higher than that of the other two groups.

### Influencing factors of effort level

In this experiment, the distribution proportion of the integration profits was the factor that most directly influenced the choice of effort level. Therefore, we performed a single-factor test on the relationship between the bidding distribution rate, the final distribution rate, the improvement rate, and the effort level. The effort level determined the integration profits. As shown in Table 2, the choices of bidding distribution rate, final distribution rate, and effort level were unstable in the different groups. However, there was a significant correlation between the improvement rate and the choice of effort level (*P<0.05*), although the correlation coefficient was slightly weak. Increasing the improvement rate will enhance the effort level of community health service centres.

**Table 2:** The relationship between the bidding distribution rate, the final distribution rate, the improvement rate, and the effort level

	The bidding distribution rate		The final distribution rate		The improvement rate	
	*r*	*P*	*r*	*P*	*r*	*P*
**Observations**	479		318		318	
**Undergraduates**	0.10	0.30	0.23	**0.04**	0.35	**<0.001**
**Postgraduates**	−0.10	0.15	0.07	0.40	0.27	**<0.001**
**Professionals**	0.14	0.14	0.26	**0.02**	0.31	**0.01**
**Total**	−0.11	**0.02**	− 0.24	0.67	0.19	**<0.001**

In addition to the distribution rate, the subject type, the contract type, the nature state, and the round of the experiment also influenced the choice of effort level, so we conducted a multifactor regression analysis. As shown in Table 3, the effort levels of the postgraduates and professionals were lower than those of the undergraduates. The greater the improvement rate was, the higher the effort level *(P < 0.001)*. The effort level with a good nature state was higher than that with a bad nature state *(P < 0.001)*.

**Table 3:** Variable dependence on effort levels within contract types

Independent variable	β	SE	Wald	df	*P*	95% CI	
						lower	upper
**The bidding distribution rate**	−0.01	0.01	0.62	1	0.43	−0.04	0.02
**The improvement rate**	0.13	0.03	20.24	1	**<0.001**	0.07	0.18
**The subject type (Reference: undergraduates)**							
**Postgraduates**	−1.50	0.24	37.85	1	**<0.001**	−1.97	− 1.02
**Professionals**	−1.71	0.30	33.56	1	**<0.001**	−2.29	−1.13
**Flexible contract (Reference: rigid contract)**	−0.32	0.22	2.18	1	0.14	−0.75	0.11
**Good nature state (Reference: bad nature state)**	0.80	0.22	12.76	1	**<0.001**	0.36	1.23
**Round (Reference: first 3 rounds)**							
**Among 4 rounds**	0.14	0.21	0.45	1	0.50	−0.28	0.57
**Final three rounds**	0.40	0.23	2.97	1	0.09	−0.06	0.86

## Discussion

### Flexible contracts were preferred by most hospital managers

The study involved students of the health management profession and professionals as subjects. We systematically analysed the relationship between the profit distribution mechanism and managers’ behaviour in the vertical integration of regional medical service systems. Hospital managers in our experiment preferred flexible contracts, with 66.39% of the subjects choosing flexible contracts. By comparison, experimental results obtained by Fehr, Hart, and Zehnder (2011) [13] in the commodity markets and the competitive environment showed that 50% of the subjects in their study chose rigid contracts and 50% chose flexible contracts, and the share of rigid contracts had an upward tendency. The information asymmetry of the medical service market is higher than that of the commodity trading market. The greater uncertainty of the medical market may lead hospital manager subjects to prefer flexible contracts. This preference reflects the requirements of autonomy.

There are two possible reasons for the preference for flexible contracts. On the one hand, the competition mechanism makes community health service centre directors strive for integration, but they can obtain contracts only with bidding distribution rates below their expectations. Therefore, they are unwilling to make the highest-level effort. On the other hand, when the nature state is poor, community health service centre directors are reluctant to make a high-level effort. In the bad state, the parties who choose the rigid contract cannot achieve a mutually beneficial transaction but must provide medical services.

### Community health service Centre directors performed shading behaviour

The most intuitive causality in our experimental design is that the effort level is related to integrating profit: the higher the effort level is, the greater the integration profit. In our study, there was no significant difference between rigid and flexible contracts *(P = 0.09).* Moreover, only 41.75% of the subjects chose the highest effort level of 10. This proves that community health service centre directors exhibit shading behaviour in the integrating process and that the answer to Research Question 1 is negative.

### Some differences could be observed between students and professionals

We found that the choices of bidding distribution rate, final distribution rate, and effort level differed between the students and professionals but that the significant correlation between the improvement rate and the effort level was similar for all the subjects. We believe that the higher bidding distribution rate and the final distribution rate of the professionals might have been related to their work experience. In addition, a market bubble study that recruited students and business professionals [27] and a medical expense payment method study that used medical students and doctors as subjects [17] both demonstrated that student subjects were similar to professionals in terms of decision-making. On the basis of the law of behaviour, we believe that our experimental results are robust.

### Higher improvement rates beyond the participants’ expectations motivated the managers’ higher cooperative behaviour

The bidding distribution rate was a reference point when the community health service centre director decided the effort level. The relationship between either the bidding distribution rate or the final distribution rate and the effort level was unstable. Thus, to some extent, the answer to Research Question 2 is also negative. However, the community health service centre directors were willing to make a greater effort when the hospital managers were willing to provide a higher improvement rate. Although there were differences in the choice of bidding distribution rate, the final distribution rate, and the effort level between the students and the professionals, their responses to the incentive mechanism were consistent, which is our most important finding.

The reason why higher improvement rates can motivate higher cooperative behaviour is, we believe, related to the theory of preference and aversion. Theories of social preferences assume that people are motivated not solely by material self-interest but also by social considerations, especially concerns about fairness [28, 29]. The community health service centre director does not choose the highest level in full accordance with the pursuit of profit maximization. The results show that community health service centre directors would perform shading behaviour when they felt that the proportion that hospital managers were providing was below their expectations. This conforms to the model of inequity aversion [30], and the behaviour is called “retaliation” in reference point theory. We hope that our experimental results prove that this direction is applicable.

Unavoidably, there are some limitations in our experimental design. One limitation is a lack of specific information about costs. The quantitative relationship between the cost and the effort level is not specified in the experimental material. Student participants may weaken the role of costs when making choices. In addition, it is unclear how much consolidated profit can be distributed in the future. This is an important factor that influences managers in decision-making. In addition, although the subjects’ interactions are anonymous, our results may have some endogeneity problems. The third limitation is that our experimental design ignores renegotiation. In reality, the competition between hospitals and community health service centres may result in renegotiation that will influence managers’ decisions. Nevertheless, the experiment still reveals the integration mechanism, and the results provide a significant reference for consummating the integration of medical systems.

## Conclusion

The manager of a medical institution is a transmitter of government policy within the institution. At the same time, the behaviour of managers has an important impact on the development of the institution. Our experiment validates the manager’s decision-making model of profit distribution in VIMDSs. Four important findings will have great significance for formulating the integrated profit distribution method or adjusting the real-life payment method of medical insurance to encourage better cooperation. In particular, higher improvement rates beyond participants’ expectation motivate higher cooperative behaviour, which will have important reference value for establishing incentive mechanisms in VIMDSs.

## Data Availability

The datasets used and/or analysed during the current study are available from the corresponding author on reasonable request.

## Abbreviations

VIMDS: Vertical Integration of Medical Delivery System.
CI: Confidence Interval
df: Degree of Freedom
K–W test: Kruskal-Wallis Test
SE: Standard Error

## Footnotes

1 The General Office of the State Council on advancing Guidance on the construction of graded clinics State Council issued [2015] No. 70, China

2 We conducted pre-experiments in the previous months, recruiting 40 undergraduates who were divided into 20 groups. Two subjects interacted in a group of one hospital manager and one centre director. The experiment showed that the first quartile of the final distribution rate was 10.00%, the third quartile was 40.00%, and the highest value was 60.00%.
